# The effect of a thumb web spacer splint on hand function in children with hemiplegic cerebral palsy

**DOI:** 10.1016/j.jtumed.2022.10.008

**Published:** 2022-11-15

**Authors:** Islam B. Ali, Fathy A. Elshazly, Mostafa S. Ali

**Affiliations:** a Faculty of Physical Therapy, Cairo University, Egypt; b Faculty of Physical Therapy, O6 University, Egypt

**Keywords:** جبيرة وظيفية, وظيفة اليد, شلل دماغي مفلوج, مساحة ويب الإبهام, جبيرة فاصل الويب, Functional splint, Hand function, Hemiplegic cerebral palsy, Thumb web space, Web spacer splint

## Abstract

**Objective:**

Many children with hemiplegic cerebral palsy (HCP) cannot maintain thumb abduction and experience obstruction caused by the thumb remaining in the palm. A web spacer splint maintains the thumb web space and opposition of the thumb for a more functional position. The aim of this study was to analyze the impact of a thumb web spacer as a functional splint on hand function in children with hemiplegic cerebral palsy.

**Methods:**

Thirty children with hemiplegic cerebral palsy (ages 4–7 years) were randomly divided into two groups (a control group and a study group). The treatment program for the control group was administered for 45 min three times/week for 8 successive weeks and the study group underwent the identical treatment regimen as the control group, as well as wearing a web spacer splint during the treatment program. Thereafter, the Peabody Developmental Motor Scale (PDMS-2) was used to assess hand function.

**Results:**

Post-treatment values in the study group demonstrated a substantial improvement in grasping and visual motor integration in the PDMS-2 when compared to the control group. Therefore, there was a significant improvement in total fine motor quotient when compared post-treatment (86.93 ± 8.94, 145.73 ± 15.04) in the control and study groups, respectively (p > 0.05).

**Conclusion:**

A web spacer splint can be a viable tool for improving hand function in children with HCP.

## الملخص

**أهداف البحث:**

لا يستطيع العديد من الأطفال المصابين بالشلل الدماغي المفلوج الحفاظ على اختطاف الإبهام كما أن الإبهام المتبقي في راحة اليد يعيقهم. تحافظ جبيرة مباعد الويب على مساحة ويب الإبهام ومقاومة الإبهام للحصول على وضع وظيفي أكثر. كان الهدف من الدراسة هو تحليل تأثير فاصل شبكة الإبهام كجبيرة وظيفية على وظيفة اليد لدى الأطفال المصابين بالشلل الدماغي المفلوج.

**طرق البحث:**

تم تقسيم ثلاثين طفلاً مصابًا بالشلل الدماغي المفلوج (تتراوح أعمارهم بين 4-7 سنوات) إلى مجموعتين بشكل عشوائي (مجموعة مراقبة ومجموعة دراسة). تمت إدارة برنامج العلاج لمجموعة التحكم لمدة 45 دقيقة 3 مرات / أسبوع لمدة 8 أسابيع متتالية وخضعت مجموعة الدراسة لنظام العلاج المماثل للمجموعة الضابطة ، بالإضافة إلى ارتداء جبيرة مباعدة الويب أثناء برنامج العلاج. بعد ذلك ، تم استخدام مقياس محرك بيبودي التنموي لتقييم وظيفة اليد.

**النتائج:**

أظهرت قيم ما بعد العلاج لمجموعة الدراسة تحسنًا كبيرًا في المتغيرات التي تم تقييمها ، حيث أظهرت مجموعة الدراسة تحسنًا كبيرًا بشكل ملحوظ في الإدراك والتكامل الحركي البصري في محرك بيبودي التنموي مقارنةً بقيم المجموعة الضابطة. لذلك ، كان هناك تحسن كبير في إجمالي الحاصل الدقيق عند مقارنة العلاج اللاحق (86.93 ± 8.94 ، 145.73 ± 15.04) في مجموعات التحكم والدراسة ، على التوالي ، مع قيمة ب < 0.05.

**الاستنتاجات:**

يمكن أن تكون جبيرة مباعد الويب أداة فعالة لتحسين وظيفة اليد بين الأطفال المصابين بالشلل الدماغي المفلوج

## Introduction

Cerebral palsy (CP) is defined as postural and movement problems that develop as a result of an insult to the developing fetal or infant brain, thus leading to the limitation of activity. CP may also be accompanied by sensory, cognitive, communication, perceptual and/or behavioral disorders and/or a seizure problem.^1^ Over the last 40 years, the incidence of CP has stayed relatively constant over time at 2.5 per 1000 live births. This is a huge disappointment because advances in perinatal care should have eliminated many occurrences of cerebral palsy by now; this could be attributable to higher survival rates for newborns who are extremely ill.^2^

Hemiplegic cerebral palsy (HCP) is the most prevalent type of CP and affects 1 in every 1300 live births.^3^ HCP is defined as unilateral motor disability caused by brain injury during pregnancy or the perinatal/postnatal periods.^4^ Heterogeneous CP is caused by conditions such as prenatal infarction, brain malformation and infection, although the most prevalent cause is perinatal ischemic stroke.^5^ In HCP, the upper limbs are usually more affected than the lower limbs and one side of the body is more significantly affected than the other.^6^

Upper limb impairments can limit a child's capacity to engage in daily tasks.^7^ The main problems and disorders of HCP include abnormal muscle tone, shoulder internal rotation, pronation of the forearm, a flexed wrist and a thumb-in-hand that make it difficult or impossible to use the upper limb and hand effectively.^8^ The thumb is a unique aspect of humans due to its position on the hand, as all useful apprehension requires thumb opposition.^9^ In HCP, the thumb-in-palm condition impairs grasp and disrupts the action of the other fingers, as well as limiting the size of the object that a child can grasp due to a lack of abduction and extension.^10^ This deformity (thumb-in-palm) can reduce the functional abilities of the hand by 50% and is thought to play a key role in limb rejection.^11^

Custom-designed physiotherapy programs have been developed to enhance muscular strength and range of motion in the affected joints.^12^ A variety of upper limb rehabilitation programs, for example, Hand Arm Intensive Bimanual Training (HABIT), Constraint-Induced Movement Therapy (CIMT), Neuro Developmental Treatment (NDT), occupational therapy, and injections of botulin toxin have been documented in children with HCP in an attempt to reduce upper limb problems and also improve hand function.^3^

A splint is an external device or cast placed on the joints and can be categorized into non-functional splints aiming to prevent contracture by providing prolonged stretch and functional splints designed to support joints in biomechanically optimal positions to improve motor task performance.^13^ A wrist and thumb splint seeks to improve the upper limb's hemiplegic pattern (wrist and finger flexion and thumb adduction) into a more functional position (neutral wrist, abduction opposition of the thumb).^14^ There is growing evidence that functional hand splints may enhance functional activity and goal achievement by providing an instant favorable influence on upper-limb skills during the execution of tasks.^15^

A web spacer splint is a functional splint formed of thermoplastic and neoprene materials. The purpose of this orthotic is to maintain the thumb in an opposing position and maintain the thumb position during different hand activities.^16^ There is growing evidence that supports the use of functional splints to improve hand function. In a previous study, Hughes et al. found that the effect size of the mean improvement in total QUEST scores for an intervention group wearing a thumb abductor splint were significantly larger than in the control group; furthermore, hand function scores decreased when the splint was removed during activities.^17^

The current study represents an innovative attempt to determine the effect of the web spacer splint as a functional splint to improve hand function in HCP. Previous studies used different types of splints that support the wrist and radio ulnar joint in addition to supporting thumb movement; however, in the present study, we only supported the thumb only during activities.

## Materials and Methods

### Study design

This was a controlled and randomized trial.

### Participants

**Figure 1:**
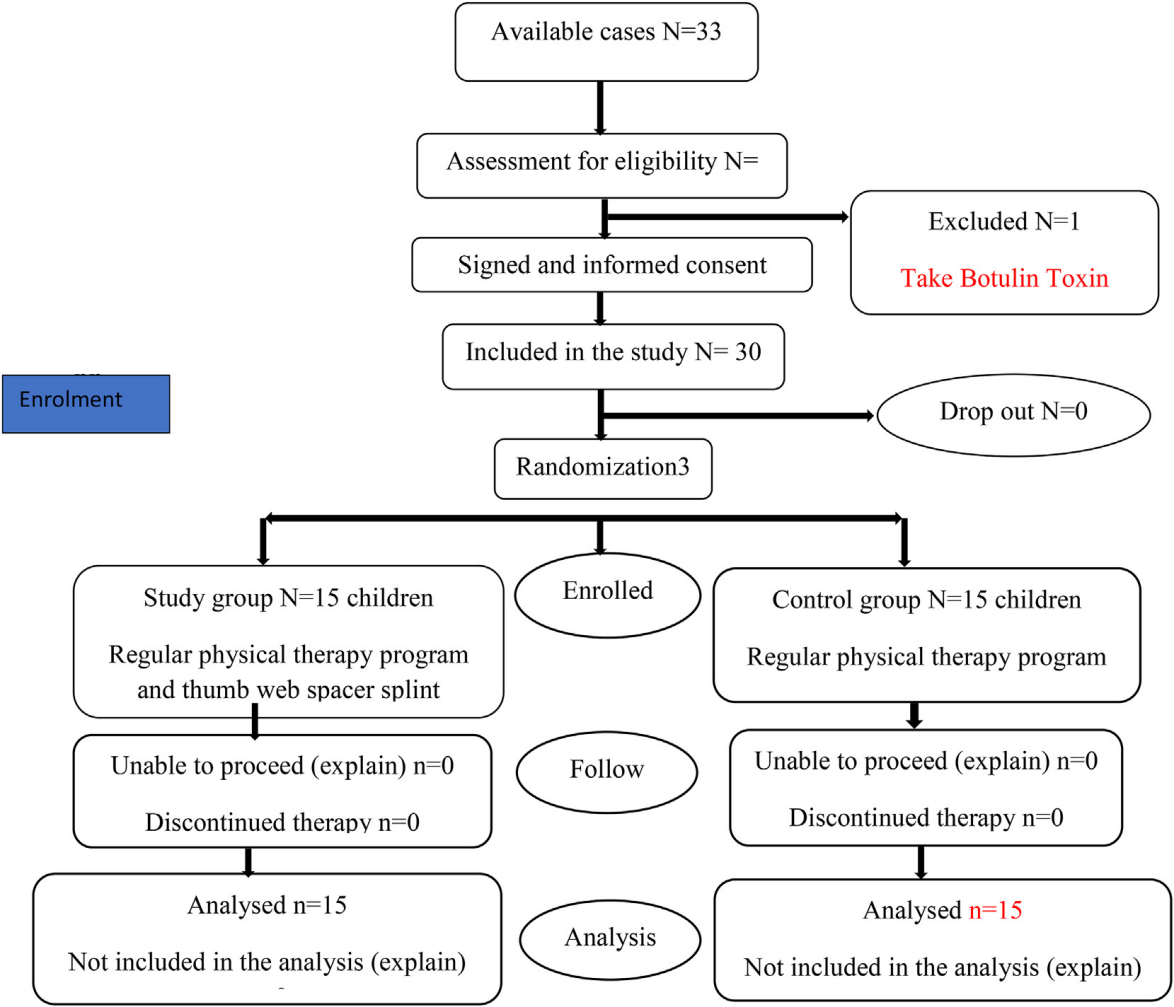
Flow chart showing the procedure used for patient randomization.

This study was conducted in February 2020 but was stopped for approximately one year due to the COVID-19 pandemic; the study continued with the practical aspect in September 2021 after the outpatient clinic of the Faculty of Physical Therapy had been re-opened. The study ended in April 2022. Thirty children with HCP (both sexes) were chosen from the occupational therapy clinic of Cairo University's Faculty of Physical Therapy and enrolled in the study. Age ranged from 4 to 7 years; please refer to the flowchart given in Figure 1. The children were split into two equal groups (Group A, the control group, and Group B, the study group). The inclusion criteria were as follows: (a) the degree of spasticity ranged from 1 to 2 according to the Modified Ashworth Scale^18^; (b) the Gross Motor Function Classification Scale^19^ was used to determine the level of function, and only children in levels I and II took part, so that they could attend treatment sessions easily and regularly and to avoid acute conditions; and (c) only children with an adducted thumb or thumb in the palm from the initial assessment were included according to patient history with a chief complain relating to hand function, and with a range of motion assessment. Those who had a previous history of hand surgery or botulin toxin injection were excluded due to potential effects on muscular function. Children with severe spasticity or bony joint limitations were also excluded as the range of motion could have been affected.

### Randomization

Simple randomization was carried out by a computer-generated randomization technique that was constructed ahead of time by the Statistical Package for Social Sciences (SPSS). Every participant was given a unique identification number that indicated which group they would be assigned to. Individually numbered index cards were sequentially inserted into non-transparent envelopes. Each participant received a hand-picked envelope and was assigned to their respective groups. The ethical committee the Faculty of Physical Therapy, Cairo University, Egypt, approved the study and a signed written consent form with parent acceptance for participation in the study and publication of the results was obtained before starting the procedure. The purpose of the study and its procedures were explained to the children's parents. Patients were chosen from the clinic and randomly divided into two groups. The control group was given a selected therapeutic program while the study group used the thumb web spacer splint during treatment sessions as well as the same therapeutic program for eight successive weeks.

### Sample size

Based on functional splint data from a pilot study conducted on five subjects in each group, sample size calculation was performed using G∗POWER statistical software (version 3.1.9.2), with a 0.05, power 80% and an effect size 1.1. The appropriate sample size for this study was determined as N 15 in each group. Post hoc analysis with a sample size of 15 in each group revealed that the power of the study was 98%.

### Outcome measurement

Hand function was measured by the Peabody Developmental Motor Scales 2nd Edition (PDMS-2).^19^ Fine motor skills (grasping and visual motor integration) were the only factors measured.

### Procedures

The Peabody Developmental Motor Scales 2nd Edition (PDMS-2)^20^ is a norm-referenced discriminative measure that is used to assess gross motor and fine motor skills in children. The gross motor scale contains 151 items divided into four subtests: reflexes, stationary, locomotion and object manipulation. The fine motor scale contains 98 items divided into grasping and visual-motor integration.

#### Fine motor quotient (TOT)

This is a combination of the results of subtests that measure the use of the small muscle systems: grasping (all ages) and visual-motor integration (all ages). We only used the fine motor scale in this study. The PDMS-2 separates fine motor skills from gross motor skills, as this scale identifies normal motor skills when using large muscles but serious problems when using small muscles.

#### Grasping

This assessment consisted of 26 items that measure a child's capacity to use his or her hands and fingers. This assessment includes manipulating movements such as grasping, releasing and reaching for items.

#### Visual-motor integration

This assessment consists of 72 items that measure a child's capacity to integrate and utilize visual perceptual skills to complete complicated eye-hand coordination tasks that include the dexterity of manipulative skills such as the handling of blocks, cups and drawing instruments.

Clinical judgment was used to select the best entry point at which the test began with items on which the child was successful. When a child received a score of 2 on three consecutive items, a base level was established. The examiner would provide more complex items until a ceiling was reached. When a child achieved 0 on three tasks in a row, the ceiling was reached. The PDMS-2 was applied to obtain the level of hand function for each child at the beginning of the study and each child was assessed again after the therapeutic program had ended. Both groups were evaluated before and after the intervention program.

### Intervention

#### Control group

For 45 min three times per week for 8 successive weeks, the 15 children were given a specific treatment program^21^ that included: (1) a stretching exercise for wrist flexors, pronators and finger flexor; (2) a strengthening exercise for shoulder flexors, shoulder abductors, elbow extensors, wrist extensors and finger extensors; (3) squeeze: children were instructed to “place your weaker arm on the table, place the ball in your hand and squeeze as hard as hard as you can for a count of 5, relax your hand for a count of 5”; (4) waiter-cup: children were instructed to “place the cup in your weaker hand, pick the cup up and move it to the 1st dot. Set the cup on the dot and place your weaker hand in your lap. Then reach with your weaker hand to pick up the cup, placing it on dot 2 etc. until you have set the cup on each dot.”; (5) chair-ups: children were instructed to “sit in a chair with both your hands on the arm rests. Using your arms NOT your legs, push your body upwards so that your bottom comes off the chair. Hold for a count of 3. Lower yourself for a count of 3”; (6) pouring water: children were instructed to “place two cups on the table, one half full of water. Hold the empty cup with your stronger hand; pick up the cup with the water using your weaker hand. Pour the water into the empty cup. Pour back for 10 times”; (7) catching and throwing balls; (8) building a tower of 10 cubes; (9) copying a line, a circle and a square, and (10) thumb opposition to touch fingers.^20^

#### Study group

A total of 15 children used the web spacer splint while performing the same specified therapeutic program as the control group, plus wearing the web spacer splint during the treatment program.

### Procedures for web spacer training

Children in the study group were asked to wear the thumb web spacer splint while performing the same designed therapeutic program.

**Figure 2:**
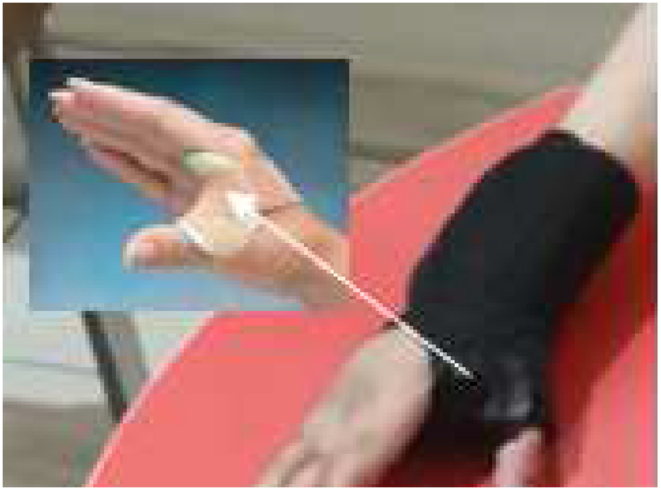
A web spacer splint.

A web spacer splint is a functional splint which is made from neoprene and thermoplastic materials. The thermoplastic part supports the thumb web space and is covered by neoprene to fit the patient's hand (see Figure 2).

### Data analysis

SPSS for Windows, version 28 was used for statistical analysis (SPSS, Inc., Chicago, IL). Data were examined for normality assumption, homogeneity of variance and the existence of extreme scores prior to final analysis. This inquiry was conducted as a requirement for performing parametric analysis of differences between groups. The independent t-test was used to determine whether the independent groups differed in terms of the dependent variable. The paired t-test was used to determine if there was a significant difference within the same group. To determine whether there was a difference in demographic variables between the two study groups before treatment, we used the unpaired t-test. The sec distribution between groups was compared by the Chi-squared test. The alpha value was set to 0.05.

## Results

### Grasping

#### Within each group

In the control group, there was a significant difference between the pre- and post-treatment mean scores of grasping. Also, in the study group, there was a significant difference between the pre- and post-treatment mean scores of grasping (see Table 1).

**Table 1:** Demographic characteristics of patients between the two groups.

	Group A $\bar{X}$ ± SD	Group B $\bar{X}$ ± SD	MD	t-value	p-value
Age (years)	6.06 ± 0.96	6.13 ± 0.91	−0.06	−0.195	0.847
Height (cm)	115.73 ± 8.19	118.26 ± 7.13	−2.53	−1.23	0.37
Weight (kg)	22.86 ± 3.33	24.23 ± 2.65	−1.37	−1.81	0.22
BMI (kg/m^2^)	17.23 ± 0.95	17.34 ± 1.42	−0.11	−0.57	0.82

$\bar{X}$: Mean, MD: Mean Difference, P-Value: Probability value, SD: Standard Deviation, t-value: Unpaired t-test.

#### Between groups

There was no significant difference in grasping pre-treatment between groups. However, there was a significant difference between groups post-treatment in favor of the study group (see Table 2).

**Table 2:** A comparison of grasping between the two groups.

	Grasping				
	Pre-treatment $\bar{X}$ ± SD	After-treatment $\bar{X}$ ± SD	P- value	Sig.	% Of change
Control group	28.93 ± 4.78	35.00 ± 5.14	0.0001	S	↑ 21%
Study group	28.60 ± 3.83	39.86 ± 4.13	0.0001	S	↑ 39.37%
P- value	0.835	0.008			

$\bar{X}$: Mean, SD: Standard Deviation, P-Value: Probability value.

### Visual motor integration

#### Within each group

In the control group, there was a significant difference between the pre- and post-treatment mean scores of visual motor integration. Also, in the study group, there was a significant difference between the pre- and post-treatment mean scores of visual motor integration.

#### Between groups

There was no significant difference between groups with regards to visual motor integration pre-treatment as (p = 0.361). However, there was a highly significant difference between groups after treatment (P = 0.00001) in favor of the study group (see Table 3).

**Table 3:** Comparison of visual motor integration between the two groups.

	Pre-treatment $\bar{X}$ ± SD	After-treatment $\bar{X}$ ± SD	P- value	% Of change
Control group	69.66 ± 9.60	86.93 ± 8.94	0.0001	↑ 24.79%
Study group	72.80 ± 8.88	105.86 ± 11.22	0.0001	↑ 45.41%
P- value	0.361	0.00001		

$\bar{X}$: Mean, SD: Standard Deviation, P-Value: Probability value.

### Fine motor quotient (TOT)

#### Within each group

In the control group, there was significant difference between the pre- and post- treatment mean TOT scores. Also, in the study group, there was significant difference between the pre- and post-treatment mean TOT scores.

#### Between groups

There was no significant difference between groups with regards to TOT pre-treatment (p = 0.895). However, there was a highly significant difference between groups after treatment (P = 0.00001 in favor of the study group) (see Table 4).

**Table 4:** Comparison of TOT between the two groups.

	Pre-treatment $\bar{X}$ ± SD	After-treatment $\bar{X}$ ± SD	P- value	% Of change
Control group	98.60 ± 14.32	121.93 ± 13.66	0.0001	↑ 23.66%
Study group	97.80 ± 18.20	145.73 ± 15.04	0.0001	↑ 49.0%
P- value	0.895	0.00001		

$\bar{X}$: Mean, SD: Standard Deviation, P-Value: Probability value.

The study group exhibited a significantly greater improvement in the PDMS-2. In the study group, there was a significant difference between the pre- and post-treatment mean TOT scores. Our findings demonstrated considerable improvements in hand function in both groups, with the study group reporting more significant improvement. These results also indicate a large impact on the variable that enhances the impact of using web a spacer splint on hand function in HCP.

## Discussion

The purpose of this study was to determine the impact of the web spacer as a functional splint to improve hand function in HCP. Analysis confirmed that hand function can be improved in HCP after a physical therapy treatment program with upper limb and hand exercises. Furthermore, our results confirm that the effect of eight weeks of using a web spacer splint during treatment sessions increased hand function activities in children with HCP.

In this study, preliminary assumption checking revealed that data for all measured variables was normally distributed, as determined by the Shapiro–Wilk test (p > 0.05). As a result, parametric statistics were employed. Children with HCP showed reduced hand function ability in terms of total scores for the fine motor quotient (TOT) in the PDMS-2 which consists of grasping and visual motor integration subtests in a controlled group and a study group (98.60 ± 14.32 & 97.80 ± 18.20, respectively).

These results of pretreatment assessment were consistent with those published previously by Alwhaibi et al.^22^ who reported that children with HCP have poor eye-hand coordination which limits activities involving the affected hand, particularly fine motor abilities such as grasping and manipulation.

In this study, children with HCP showed an increase in hand function ability in terms of the total score in both groups; scores in the control group increased from 98.60 ± 14.32 to 121.93 ± 13.66 and the study group increased from 97.80 ± 18.20 to 145.73 ± 15.04, respectively. The improvement in the study group was much greater.

The observed improvement in the two groups could be attributable to the impact of the specified program as prescribed, which included stretching and strengthening exercises for the shoulder, elbow, hand and fingers to increase muscle strength and range of motion. We used a variety of hand exercises to improve hand function by using different sizes of blocks, cups, balls, cubes, pens and papers.

These findings agree with those of Choudhary et al.^23^ who found that a 4-week course of “Modified Constraint Induced Movement Therapy” improved the upper limb abilities in children with HCP. The improvements in upper limb capabilities lasted for 8 weeks after the intervention was stopped. In a previous study, Gordon et al.^24^ found that bimanual hand use can be improved by the application of bimanual training. This implies that training specificity is compatible with motor learning theories as children with HCP showed improvement in all tests. Thus, a physical therapy program can play a key role in improving hand function in children with HCP. However, the study group showed more improvement following the use of a web spacer splint. Our findings are reinforced by those of Louwers et al.^14^ who demonstrated the effects of a wrist and thumb splint on bimanual hand function in 25 children with HCP aged 4–11 years. Wearing the brace improved results in the Assisting Hand Assessment (which assesses the automatic use of the affected hand to help in activities which require using both hands) by about 3.2 raw scores; approximately half of the children improved by more than 4 points.

It is possible that the study group's larger improvement was due to using the thumb web spacer. Considering the observed increase in PDMS-2 score, we hypothesize that changes in hand function could be improved by using functional splints. Our results agreed with those of Berge et al.^25^ who suggested that thumb opponent splints may help children with HCP to improve their hand function. Improvements in treatment goal achievement following splint use suggests that a positive impulse was given the hand function of children by using a web spacer splint. Furthermore, the increase in hand function and the increase in PDMS-2 score observed in the study group following the application of a web spacer splint might be due to the provision of support to joints in biomechanically optimal positions during hand activities; these findings concurred with those of Basu.^26^ We concluded that the use of a web spacer splint improves hand function; these findings were in line with those of Jensen et al.^27^ who found a strong correlation between the use of a web spacer splint and hand function. Moreover, using a thumb web spacer and the prescribed physiotherapy program appears to have influenced the improvement in hand function that appeared in the study group. Thus, we confirmed that the use of a web spacer splint improves hand function in children with HCP. This study lasted only 8 weeks; we recommend conducting a longer-term study to validate the long-term impact of this treatment strategy.

Finally, the current findings indicate that employing a web spacer splint in conjunction with a physical therapy program should be a useful option to enhance hand function in children with HCP.

### Study limitations

1. The practical aspect of this study was delayed as the outpatient clinic of the Faculty of Physical Therapy closed for approximately one year as a result of the COVID-19 pandemic; we only started the practical component in September 2021.
2. The effect of psychological factors which may reflect on the treatment program were not evaluated.

## Conclusion

The current study found that both the study and control groups improved significantly in terms of hand function and PDMS-2 score after the application of a designed physical therapy program that included stretching, strengthening exercises for the shoulder, elbow, hand, and fingers and a variety of hand exercises; however, there was more improvement in favor of the study group. Thus, using a web spacer splint can efficiently improve hand function in children with HCP, thus suggesting that the web spacer is a useful functional splint for improving hand function in children with HCP.

## Recommendations

This study showed that using a web spacer splint can efficiently improve hand function in HCP. Thus, for such children, a thumb web spacer should be prescribed as part of a rehabilitation program. The findings of this study may provide assistance to individuals with HCP who suffer from hand impairments such as “Thumb in Palm Deformity” to improve hand function.

## Source of funding

This study received no specific funding from public, commercial, or not-for-profit funding organizations.

## Conflict of interest

The authors have no conflict of interest to declare.

## Ethical approval

This study was approved by the Ethical Committee of the faculty of Physical Therapy, Cairo University (NO: P.T.REC/012/008545) on April 1st, 2021.

## Authors contributions

IBA conceived and designed the study, conducted the research, provided research materials, and collected and organized the data. FAS and MSM analyzed and interpreted data, wrote the initial and final drafts of the article, and provided logistical support. All authors have critically reviewed and approved the final draft and are responsible for the content and similarity index of the manuscript.
